# [2-({Benz­yl[2-(benz­yl{5-methyl-2-oxido-3-[(pyridin-2-ylmeth­yl)imino­meth­yl]benz­yl}amino)­eth­yl]aza­nium­yl}meth­yl)-4-methyl-6-[(pyridin-2-ylmeth­yl)imino­meth­yl]phenolato]nickel(II) perchlorate methanol disolvate

**DOI:** 10.1107/S1600536812024191

**Published:** 2012-05-31

**Authors:** Ming Liu, Jia-Wei Mao, Zhi-Quan Pan

**Affiliations:** a Key Laboratory for Green Chemical Process of the Ministry of Education, Wuhan Institute of Technology, Wuhan 430073, People’s Republic of China; b College of Chemistry and Molecular Sciences, Wuhan University, Wuhan 430072, People’s Republic of China

## Abstract

In the solvated title complex, [Ni(C_46_H_47_N_6_O_2_)]ClO_4_·2CH_4_O, the coordination sphere around the Ni^II^ ion can be described as distorted *cis*-NiO_2_N_4_ octa­hedral defined by two phenolate O atoms and four N atoms from the hexa­dentate ligand. An intra­molecular bifurcated N—H⋯(N,O) hydrogen bond helps to establish the conformation of the complex mol­ecule. In the crystal, the components are connected by O—H⋯O and C—H⋯O hydrogen bonds.

## Related literature

For related complexes, see: Choi *et al.* (1999 ▶); Golchoubian *et al.* (2007*a* ▶,*b* ▶, 2010 ▶, 2012 ▶); Pan *et al.* (2011 ▶). For the preparation of the ligand, see: Ding *et al.* (2012 ▶). 
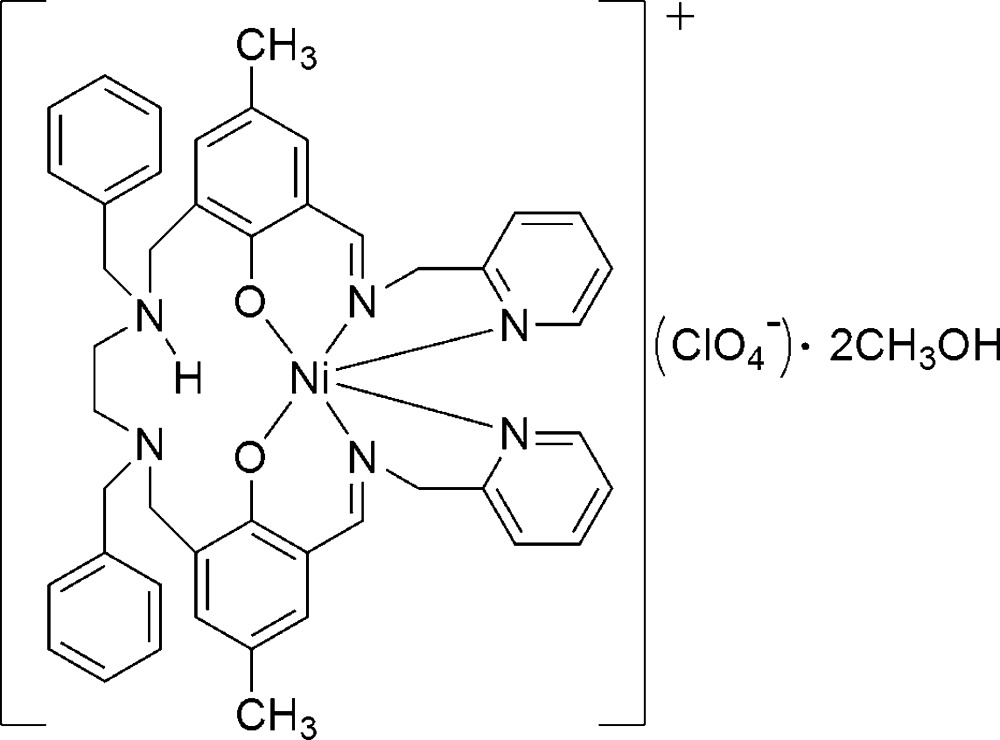


## Experimental

### 

#### Crystal data

[Ni(C_46_H_47_N_6_O_2_)]ClO_4_·2CH_4_O
*M* *_r_* = 938.14
Triclinic, 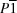

*a* = 11.2875 (6) Å
*b* = 13.0874 (7) Å
*c* = 16.3325 (9) Å
α = 93.902 (1)°
β = 107.537 (1)°
γ = 95.310 (1)°
*V* = 2279.0 (2) Å^3^
*Z* = 2
Mo *K*α radiation
μ = 0.55 mm^−1^
*T* = 100 K
0.16 × 0.12 × 0.10 mm

#### Data collection

Bruker APEXII CCD diffractometer
Absorption correction: multi-scan (*SADABS*; Bruker, 2000 ▶) *T*_min_ = 0.918, *T*_max_ = 0.948
13755 measured reflections
8368 independent reflections
7377 reflections with *I* > 2σ(*I*)
*R*_int_ = 0.016

#### Refinement

*R*[*F* ^2^ > 2σ(*F* ^2^)] = 0.037
*wR*(*F* ^2^) = 0.165
*S* = 1.15
8368 reflections
586 parameters
H atoms treated by a mixture of independent and constrained refinement
Δρ_max_ = 0.65 e Å^−3^
Δρ_min_ = −0.57 e Å^−3^

### 

Data collection: *APEX2* (Bruker, 2000 ▶); cell refinement: *SAINT* (Bruker, 2000 ▶); data reduction: *SAINT*; program(s) used to solve structure: *SHELXS97* (Sheldrick, 2008 ▶); program(s) used to refine structure: *SHELXL97* (Sheldrick, 2008 ▶); molecular graphics: *SHELXTL* (Sheldrick, 2008 ▶); software used to prepare material for publication: *SHELXTL*.

## Supplementary Material

Crystal structure: contains datablock(s) global, I. DOI: 10.1107/S1600536812024191/hb6819sup1.cif

Structure factors: contains datablock(s) I. DOI: 10.1107/S1600536812024191/hb6819Isup2.hkl

Additional supplementary materials:  crystallographic information; 3D view; checkCIF report

## Figures and Tables

**Table 1:** Selected bond lengths (Å)

Ni1—N5	2.022 (2)
Ni1—N2	2.024 (2)
Ni1—N6	2.123 (2)
Ni1—N1	2.135 (2)
Ni1—O1	2.0277 (16)
Ni1—O2	2.0543 (16)

**Table 2:** Hydrogen-bond geometry (Å, °)

*D*—H⋯*A*	*D*—H	H⋯*A*	*D*⋯*A*	*D*—H⋯*A*
N4—H4*A*⋯O2	0.81 (3)	2.21 (3)	2.829 (3)	134 (3)
N4—H4*A*⋯N3	0.81 (3)	2.35 (3)	2.809 (3)	117 (2)
C45—H45⋯O8^i^	0.95	2.52	3.417 (3)	156
C41—H41*A*⋯O6	0.99	2.34	3.276 (3)	157
C31—H31⋯O8^ii^	0.95	2.43	3.365 (3)	167
C30—H30⋯O5^ii^	0.95	2.58	3.524 (3)	171
C28—H28⋯O6^iii^	0.95	2.56	3.503 (3)	173
C7—H7⋯O6^i^	0.95	2.60	3.289 (3)	130
C4—H4⋯O4^iv^	0.95	2.49	3.405 (3)	163
O8—H8⋯O3^v^	0.84	1.91	2.748 (3)	176
O3—H3*A*⋯O1^vi^	0.84	1.91	2.723 (3)	164

Symmetry codes: (i) 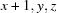; (ii) 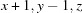; (iii) 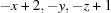; (iv) 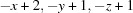; (v) 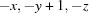; (vi) 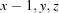.

**Fig. 1.**
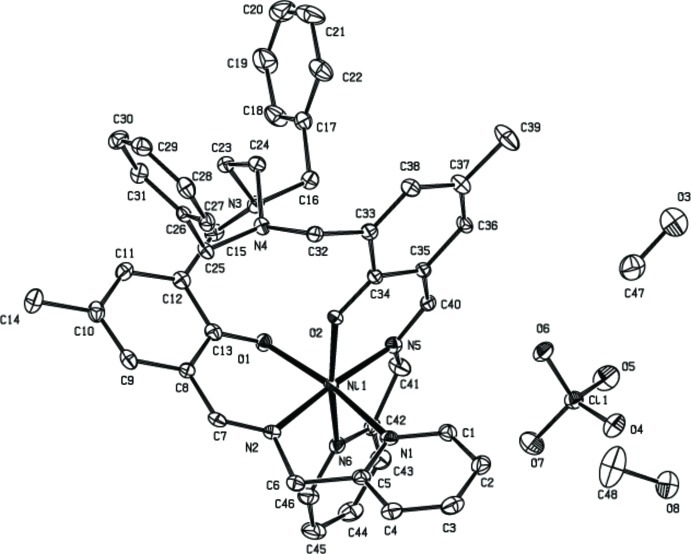
Molecular structure of the title compound. Displacement ellipsoids are drawn at the 30% probability level. H atoms have been omitted for clarity.

## supplementary crystallographic information

### Comment

Recently, study of Schiff base complexes with macrocycle ligands has been given considerable attention because of their interesting biochemical properties (Choi *et al.*, 1999; Golchoubian *et al.*, 2007*a*,*b*; Golchoubian *et al.*, 2010; Golchoubian *et al.*, 2012). In this paper, we report on the the synthesis and crystal structure of the title compound, a new nickel(II) complex obtained by the reaction of 3,3'-(ethane-1,2-diylbis(benzylazanediyl)bis(methylene) bis(2-hydroxy-5-methylbenzaldehyde)(*L^1^*)and 2-(Aminomethyl) pyridine (*L^2^*) in the presence of Ni(ClO_4_)_2_.6H_2_O.The coordination geometry for central Ni^II^ atom can be described as distorted octahedral and the basal bond distances around the Ni atom are in the range of 2.022-2.135 Å (Fig.1, Tab. 1), and the distances of amino N to Ni are shorter than those of pyridine N to Ni.

### Experimental

The ligand L^1^ was prepared according to the literature method (Ding *et al.*, 2012). L^2^ (0.0432 g, 0.40 mmol) dissolved in sbsolute methanol (10 ml) was added dropwise to a solution of L^1^ (0.1072 g, 0.20 mmol). The mixture was stirred for 6h and then an absolute methanol solution of Ni(ClO_4_)_2_.6H_2_O (0.0731 g, 0.20 mmol) was added dropwise. The mixture was stirred at room temperature for 8 h and filtered. Orange blocks were obtained by evaporation of the filtrate at room temperature for three weeks.

### Refinement

All C-bound H atoms were placed in calculated positions with 0.93–0.97 Å, and included in the refinement in the riding-model approximation, with *U*(H) set to 1.2–1.5*U*_eq_(C).

### Crystal data

**Table:**

[Ni(C_46_H_47_N_6_O_2_)]ClO_4_·2CH_4_O	*Z* = 2
*M**_r_* = 938.14	*F*(000) = 988
Triclinic, *P*1	*D*_x_ = 1.367 Mg m^−^^3^
*a* = 11.2875 (6) Å	Mo *K*α radiation, λ = 0.71073 Å
*b* = 13.0874 (7) Å	Cell parameters from 9531 reflections
*c* = 16.3325 (9) Å	θ = 2.2–31.7°
α = 93.902 (1)°	µ = 0.55 mm^−^^1^
β = 107.537 (1)°	*T* = 100 K
γ = 95.310 (1)°	Block, orange
*V* = 2279.0 (2) Å^3^	0.16 × 0.12 × 0.10 mm

### Data collection

**Table:**

Bruker APEXII CCD diffractometer	8368 independent reflections
Radiation source: fine-focus sealed tube	7377 reflections with *I* > 2σ(*I*)
Graphite monochromator	*R*_int_ = 0.016
φ and ω scans	θ_max_ = 25.5°, θ_min_ = 1.3°
Absorption correction: multi-scan (*SADABS*; Bruker, 2000)	*h* = −12→13
*T*_min_ = 0.918, *T*_max_ = 0.948	*k* = −15→12
13755 measured reflections	*l* = −19→19

### Refinement

**Table:**

Refinement on *F*^2^	Primary atom site location: structure-invariant direct methods
Least-squares matrix: full	Secondary atom site location: difference Fourier map
*R*[*F*^2^ > 2σ(*F*^2^)] = 0.037	Hydrogen site location: inferred from neighbouring sites
*wR*(*F*^2^) = 0.165	H atoms treated by a mixture of independent and constrained refinement
*S* = 1.15	*w* = 1/[σ^2^(*F*_o_^2^) + (0.1196*P*)^2^ + 0.2909*P*] where *P* = (*F*_o_^2^ + 2*F*_c_^2^)/3
8368 reflections	(Δ/σ)_max_ = 0.004
586 parameters	Δρ_max_ = 0.65 e Å^−^^3^
0 restraints	Δρ_min_ = −0.57 e Å^−^^3^

### Special details

**Table:**

Geometry. All esds (except the esd in the dihedral angle between two l.s. planes) are estimated using the full covariance matrix. The cell esds are taken into account individually in the estimation of esds in distances, angles and torsion angles; correlations between esds in cell parameters are only used when they are defined by crystal symmetry. An approximate (isotropic) treatment of cell esds is used for estimating esds involving l.s. planes.
Refinement. Refinement of F^2^ against ALL reflections. The weighted R-factor wR and goodness of fit S are based on F^2^, conventional R-factors R are based on F, with F set to zero for negative F^2^. The threshold expression of F^2^ > 2sigma(F^2^) is used only for calculating R-factors(gt) etc. and is not relevant to the choice of reflections for refinement. R-factors based on F^2^ are statistically about twice as large as those based on F, and R- factors based on ALL data will be even larger.

### Fractional atomic coordinates and isotropic or equivalent isotropic displacement parameters (Å^2^)

**Table:**

	*x*	*y*	*z*	*U*_iso_*/*U*_eq_	
Ni1	1.00784 (2)	0.23501 (2)	0.303149 (17)	0.01176 (13)	
C1	0.9500 (2)	0.33199 (18)	0.46596 (16)	0.0171 (5)	
H1	0.8651	0.3067	0.4363	0.021*	
C2	0.9795 (2)	0.37976 (19)	0.54881 (17)	0.0213 (5)	
H2	0.9163	0.3869	0.5756	0.026*	
C3	1.1036 (3)	0.4172 (2)	0.59222 (17)	0.0230 (6)	
H3	1.1270	0.4505	0.6492	0.028*	
C4	1.1926 (2)	0.40475 (19)	0.55063 (16)	0.0191 (5)	
H4	1.2781	0.4297	0.5790	0.023*	
C5	1.1564 (2)	0.35590 (18)	0.46758 (15)	0.0157 (5)	
C6	1.2490 (2)	0.34329 (19)	0.41852 (15)	0.0169 (5)	
H6A	1.2703	0.4100	0.3982	0.020*	
H6B	1.3268	0.3225	0.4574	0.020*	
C7	1.2743 (2)	0.21904 (18)	0.31576 (15)	0.0145 (5)	
H7	1.3611	0.2390	0.3436	0.017*	
C8	1.2389 (2)	0.13883 (17)	0.24411 (15)	0.0134 (5)	
C9	1.3367 (2)	0.08859 (19)	0.22811 (16)	0.0168 (5)	
H9	1.4205	0.1128	0.2614	0.020*	
C10	1.3157 (2)	0.00611 (19)	0.16641 (16)	0.0182 (5)	
C11	1.1905 (2)	−0.03080 (18)	0.12067 (15)	0.0165 (5)	
H11	1.1734	−0.0894	0.0794	0.020*	
C12	1.0913 (2)	0.01575 (18)	0.13388 (14)	0.0143 (5)	
C13	1.1126 (2)	0.10685 (18)	0.19223 (14)	0.0141 (5)	
C14	1.4211 (3)	−0.0465 (2)	0.14902 (18)	0.0263 (6)	
H14A	1.5016	−0.0084	0.1833	0.039*	
H14B	1.4133	−0.0479	0.0875	0.039*	
H14C	1.4164	−0.1173	0.1650	0.039*	
C15	0.9571 (2)	−0.03342 (18)	0.09406 (14)	0.0157 (5)	
H15A	0.9022	0.0196	0.0714	0.019*	
H15B	0.9511	−0.0857	0.0458	0.019*	
C16	0.7835 (2)	−0.08818 (19)	0.15335 (15)	0.0161 (5)	
H16A	0.7544	−0.0215	0.1361	0.019*	
H16B	0.7728	−0.0971	0.2105	0.019*	
C17	0.7005 (2)	−0.17363 (19)	0.08890 (16)	0.0171 (5)	
C18	0.6706 (2)	−0.1667 (2)	0.00026 (17)	0.0247 (6)	
H18	0.6968	−0.1048	−0.0199	0.030*	
C19	0.6028 (3)	−0.2491 (3)	−0.05936 (19)	0.0346 (7)	
H19	0.5848	−0.2436	−0.1195	0.042*	
C20	0.5627 (3)	−0.3376 (2)	−0.0310 (2)	0.0356 (7)	
H20	0.5163	−0.3935	−0.0715	0.043*	
C21	0.5894 (3)	−0.3461 (2)	0.0569 (2)	0.0355 (7)	
H21	0.5614	−0.4078	0.0765	0.043*	
C22	0.6578 (2)	−0.2633 (2)	0.11643 (18)	0.0252 (6)	
H22	0.6751	−0.2689	0.1765	0.030*	
C23	0.9709 (2)	−0.17928 (18)	0.18041 (14)	0.0142 (5)	
H23A	1.0501	−0.1778	0.1659	0.017*	
H23B	0.9119	−0.2373	0.1441	0.017*	
C24	0.9957 (2)	−0.19542 (17)	0.27522 (14)	0.0141 (5)	
H24A	0.9155	−0.2063	0.2881	0.017*	
H24B	1.0396	−0.2573	0.2884	0.017*	
C25	1.2124 (2)	−0.09751 (18)	0.33744 (15)	0.0142 (5)	
H25A	1.2226	−0.0927	0.2797	0.017*	
H25B	1.2578	−0.0344	0.3747	0.017*	
C26	1.2696 (2)	−0.19011 (18)	0.37490 (14)	0.0130 (5)	
C27	1.3107 (2)	−0.19528 (19)	0.46436 (15)	0.0158 (5)	
H27	1.3026	−0.1397	0.5021	0.019*	
C28	1.3631 (2)	−0.2814 (2)	0.49803 (16)	0.0187 (5)	
H28	1.3897	−0.2851	0.5586	0.022*	
C29	1.3766 (2)	−0.3624 (2)	0.44274 (17)	0.0198 (5)	
H29	1.4121	−0.4214	0.4655	0.024*	
C30	1.3380 (2)	−0.3563 (2)	0.35439 (17)	0.0207 (5)	
H30	1.3483	−0.4111	0.3168	0.025*	
C31	1.2845 (2)	−0.27092 (19)	0.32042 (15)	0.0172 (5)	
H31	1.2580	−0.2677	0.2597	0.021*	
C32	1.0519 (2)	−0.08714 (18)	0.41611 (14)	0.0136 (5)	
H32A	1.0554	−0.1529	0.4429	0.016*	
H32B	1.1173	−0.0351	0.4552	0.016*	
C33	0.9263 (2)	−0.05175 (18)	0.40261 (14)	0.0139 (5)	
C34	0.9098 (2)	0.04446 (18)	0.36746 (14)	0.0126 (5)	
C35	0.7850 (2)	0.07242 (18)	0.34375 (14)	0.0127 (5)	
C36	0.6904 (2)	0.00995 (18)	0.36406 (15)	0.0151 (5)	
H36	0.6088	0.0307	0.3494	0.018*	
C37	0.7102 (2)	−0.0785 (2)	0.40363 (16)	0.0188 (5)	
C38	0.8306 (2)	−0.11052 (19)	0.42039 (15)	0.0158 (5)	
H38	0.8462	−0.1739	0.4444	0.019*	
C39	0.6089 (3)	−0.1415 (2)	0.4280 (2)	0.0320 (7)	
H39A	0.6327	−0.1407	0.4909	0.048*	
H39B	0.5984	−0.2127	0.4019	0.048*	
H39C	0.5299	−0.1119	0.4070	0.048*	
C40	0.7457 (2)	0.15415 (18)	0.29014 (15)	0.0142 (5)	
H40	0.6592	0.1622	0.2725	0.017*	
C41	0.7587 (2)	0.2837 (2)	0.19932 (17)	0.0208 (5)	
H41A	0.6863	0.3092	0.2132	0.025*	
H41B	0.7273	0.2435	0.1419	0.025*	
C42	0.8506 (2)	0.37440 (18)	0.19686 (15)	0.0160 (5)	
C43	0.8096 (2)	0.45523 (19)	0.14941 (17)	0.0213 (5)	
H43	0.7228	0.4576	0.1223	0.026*	
C44	0.8968 (3)	0.5321 (2)	0.14216 (17)	0.0243 (6)	
H44	0.8708	0.5878	0.1094	0.029*	
C45	1.0227 (3)	0.5273 (2)	0.18317 (17)	0.0247 (6)	
H45	1.0845	0.5793	0.1788	0.030*	
C46	1.0565 (2)	0.44504 (19)	0.23069 (16)	0.0194 (5)	
H46	1.1427	0.4419	0.2593	0.023*	
C47	0.0874 (3)	0.3074 (2)	0.05663 (19)	0.0343 (7)	
H47A	0.0787	0.3767	0.0380	0.051*	
H47B	0.1256	0.2682	0.0201	0.051*	
H47C	0.1407	0.3128	0.1169	0.051*	
C48	0.2841 (4)	0.6376 (3)	0.0905 (2)	0.0536 (10)	
H48A	0.3329	0.6732	0.0581	0.080*	
H48B	0.2415	0.5719	0.0577	0.080*	
H48C	0.3402	0.6243	0.1465	0.080*	
Cl1	0.50194 (5)	0.42157 (4)	0.28405 (4)	0.01736 (16)	
N1	1.03570 (18)	0.31955 (15)	0.42526 (13)	0.0145 (4)	
N2	1.19670 (18)	0.26522 (15)	0.34448 (12)	0.0137 (4)	
N3	0.91831 (18)	−0.08255 (15)	0.16252 (12)	0.0145 (4)	
N4	1.07460 (18)	−0.10230 (16)	0.32941 (12)	0.0129 (4)	
H4A	1.047 (3)	−0.053 (2)	0.3067 (18)	0.016*	
N5	0.81864 (18)	0.21704 (15)	0.26433 (13)	0.0153 (4)	
N6	0.97235 (18)	0.36934 (15)	0.23810 (13)	0.0144 (4)	
O1	1.01817 (15)	0.15622 (13)	0.19471 (10)	0.0147 (3)	
O2	1.00587 (14)	0.09689 (12)	0.35570 (10)	0.0130 (3)	
O3	−0.03307 (19)	0.25596 (17)	0.04924 (13)	0.0338 (5)	
H3A	−0.0264	0.2158	0.0880	0.051*	
O4	0.51719 (17)	0.45978 (15)	0.37212 (12)	0.0253 (4)	
O5	0.37375 (18)	0.42248 (16)	0.23252 (13)	0.0309 (5)	
O6	0.53263 (17)	0.31694 (14)	0.28219 (12)	0.0232 (4)	
O7	0.5843 (2)	0.48434 (16)	0.25082 (14)	0.0343 (5)	
O8	0.19526 (19)	0.69926 (16)	0.10333 (12)	0.0293 (4)	
H8	0.1481	0.7116	0.0553	0.044*	

### Atomic displacement parameters (Å^2^)

**Table:**

	*U*^11^	*U*^22^	*U*^33^	*U*^12^	*U*^13^	*U*^23^
Ni1	0.00991 (19)	0.01058 (19)	0.01500 (19)	0.00069 (12)	0.00415 (13)	0.00207 (12)
C1	0.0166 (12)	0.0138 (11)	0.0225 (12)	0.0019 (9)	0.0078 (10)	0.0043 (9)
C2	0.0287 (14)	0.0149 (12)	0.0249 (13)	0.0045 (10)	0.0142 (11)	0.0041 (10)
C3	0.0333 (15)	0.0163 (12)	0.0189 (12)	0.0009 (11)	0.0082 (11)	−0.0007 (10)
C4	0.0199 (12)	0.0148 (12)	0.0197 (12)	0.0006 (10)	0.0029 (10)	−0.0007 (9)
C5	0.0159 (12)	0.0105 (11)	0.0211 (12)	0.0016 (9)	0.0060 (10)	0.0033 (9)
C6	0.0144 (11)	0.0161 (12)	0.0187 (12)	−0.0022 (9)	0.0045 (9)	−0.0007 (9)
C7	0.0129 (11)	0.0127 (11)	0.0171 (11)	−0.0019 (9)	0.0040 (9)	0.0044 (9)
C8	0.0144 (11)	0.0118 (11)	0.0150 (11)	0.0011 (9)	0.0061 (9)	0.0023 (9)
C9	0.0129 (11)	0.0192 (12)	0.0207 (12)	0.0036 (9)	0.0066 (9)	0.0089 (10)
C10	0.0203 (13)	0.0205 (12)	0.0181 (12)	0.0060 (10)	0.0104 (10)	0.0070 (10)
C11	0.0251 (13)	0.0142 (11)	0.0134 (11)	0.0043 (10)	0.0094 (10)	0.0032 (9)
C12	0.0156 (12)	0.0149 (11)	0.0128 (11)	0.0031 (9)	0.0038 (9)	0.0062 (9)
C13	0.0161 (12)	0.0139 (11)	0.0140 (11)	0.0017 (9)	0.0060 (9)	0.0066 (9)
C14	0.0263 (14)	0.0285 (15)	0.0268 (14)	0.0094 (12)	0.0114 (11)	−0.0010 (11)
C15	0.0191 (12)	0.0153 (11)	0.0111 (11)	0.0013 (9)	0.0026 (9)	0.0011 (9)
C16	0.0153 (11)	0.0174 (12)	0.0151 (11)	0.0031 (9)	0.0036 (9)	0.0010 (9)
C17	0.0112 (11)	0.0189 (12)	0.0200 (12)	0.0026 (9)	0.0029 (9)	0.0007 (10)
C18	0.0189 (13)	0.0309 (15)	0.0203 (13)	−0.0066 (11)	0.0034 (10)	0.0020 (11)
C19	0.0232 (15)	0.0479 (19)	0.0261 (15)	−0.0091 (13)	0.0044 (12)	−0.0080 (13)
C20	0.0218 (14)	0.0304 (16)	0.0445 (18)	−0.0028 (12)	0.0011 (13)	−0.0147 (13)
C21	0.0168 (13)	0.0218 (14)	0.062 (2)	−0.0021 (11)	0.0038 (13)	0.0095 (14)
C22	0.0150 (12)	0.0275 (14)	0.0314 (14)	0.0011 (11)	0.0031 (11)	0.0110 (11)
C23	0.0144 (11)	0.0134 (11)	0.0140 (11)	0.0017 (9)	0.0035 (9)	0.0008 (9)
C24	0.0131 (11)	0.0145 (12)	0.0139 (11)	0.0008 (9)	0.0030 (9)	0.0021 (9)
C25	0.0103 (11)	0.0163 (12)	0.0167 (11)	0.0002 (9)	0.0050 (9)	0.0043 (9)
C26	0.0095 (10)	0.0148 (11)	0.0142 (11)	−0.0001 (9)	0.0035 (9)	0.0012 (9)
C27	0.0121 (11)	0.0190 (12)	0.0164 (11)	0.0026 (9)	0.0045 (9)	0.0013 (9)
C28	0.0120 (11)	0.0248 (13)	0.0192 (12)	0.0039 (10)	0.0033 (9)	0.0074 (10)
C29	0.0131 (12)	0.0178 (12)	0.0262 (13)	0.0021 (10)	0.0023 (10)	0.0044 (10)
C30	0.0171 (12)	0.0169 (12)	0.0256 (13)	0.0043 (10)	0.0030 (10)	−0.0024 (10)
C31	0.0141 (11)	0.0223 (13)	0.0136 (11)	0.0037 (10)	0.0018 (9)	−0.0005 (9)
C32	0.0167 (12)	0.0137 (11)	0.0101 (10)	−0.0005 (9)	0.0048 (9)	0.0002 (8)
C33	0.0131 (11)	0.0168 (12)	0.0093 (10)	−0.0014 (9)	0.0016 (9)	−0.0019 (9)
C34	0.0121 (11)	0.0146 (11)	0.0103 (10)	−0.0018 (9)	0.0039 (8)	−0.0012 (8)
C35	0.0129 (11)	0.0131 (11)	0.0118 (10)	−0.0007 (9)	0.0044 (9)	−0.0008 (9)
C36	0.0090 (11)	0.0196 (12)	0.0150 (11)	−0.0023 (9)	0.0029 (9)	−0.0013 (9)
C37	0.0149 (12)	0.0216 (13)	0.0196 (12)	−0.0013 (10)	0.0053 (10)	0.0048 (10)
C38	0.0169 (12)	0.0159 (12)	0.0145 (11)	0.0015 (9)	0.0041 (9)	0.0057 (9)
C39	0.0195 (14)	0.0338 (16)	0.0476 (18)	0.0034 (12)	0.0135 (13)	0.0230 (14)
C40	0.0103 (11)	0.0140 (11)	0.0173 (11)	0.0015 (9)	0.0035 (9)	−0.0016 (9)
C41	0.0158 (12)	0.0189 (13)	0.0264 (13)	0.0005 (10)	0.0037 (10)	0.0077 (10)
C42	0.0183 (12)	0.0135 (11)	0.0184 (12)	0.0046 (9)	0.0080 (10)	0.0030 (9)
C43	0.0222 (13)	0.0175 (12)	0.0249 (13)	0.0064 (10)	0.0063 (10)	0.0059 (10)
C44	0.0356 (15)	0.0143 (12)	0.0235 (13)	0.0054 (11)	0.0080 (11)	0.0072 (10)
C45	0.0318 (15)	0.0176 (13)	0.0242 (13)	−0.0052 (11)	0.0102 (11)	0.0036 (10)
C46	0.0204 (13)	0.0166 (12)	0.0189 (12)	−0.0029 (10)	0.0046 (10)	−0.0005 (10)
C47	0.0465 (19)	0.0332 (16)	0.0260 (14)	0.0032 (14)	0.0161 (13)	0.0029 (12)
C48	0.058 (2)	0.076 (3)	0.0326 (17)	0.038 (2)	0.0137 (16)	0.0063 (17)
Cl1	0.0176 (3)	0.0162 (3)	0.0180 (3)	0.0022 (2)	0.0050 (2)	0.0016 (2)
N1	0.0155 (10)	0.0105 (9)	0.0174 (10)	0.0002 (8)	0.0048 (8)	0.0025 (8)
N2	0.0145 (10)	0.0109 (9)	0.0153 (9)	−0.0005 (8)	0.0044 (8)	0.0016 (8)
N3	0.0142 (10)	0.0144 (10)	0.0147 (9)	0.0039 (8)	0.0035 (8)	0.0023 (8)
N4	0.0118 (10)	0.0152 (10)	0.0124 (9)	0.0033 (8)	0.0035 (8)	0.0048 (8)
N5	0.0150 (10)	0.0140 (10)	0.0170 (10)	0.0016 (8)	0.0053 (8)	0.0017 (8)
N6	0.0165 (10)	0.0119 (10)	0.0169 (10)	0.0037 (8)	0.0073 (8)	0.0026 (8)
O1	0.0122 (8)	0.0169 (8)	0.0143 (8)	0.0025 (7)	0.0032 (6)	0.0000 (6)
O2	0.0112 (8)	0.0117 (8)	0.0162 (8)	−0.0007 (6)	0.0053 (6)	0.0010 (6)
O3	0.0344 (12)	0.0415 (13)	0.0249 (10)	0.0073 (10)	0.0050 (9)	0.0146 (9)
O4	0.0289 (10)	0.0260 (10)	0.0207 (9)	0.0090 (8)	0.0061 (8)	0.0002 (7)
O5	0.0221 (10)	0.0337 (11)	0.0280 (10)	0.0077 (9)	−0.0053 (8)	−0.0030 (8)
O6	0.0284 (10)	0.0164 (9)	0.0267 (10)	0.0056 (7)	0.0113 (8)	−0.0009 (7)
O7	0.0406 (12)	0.0279 (11)	0.0410 (12)	−0.0022 (9)	0.0234 (10)	0.0088 (9)
O8	0.0315 (11)	0.0361 (11)	0.0223 (9)	0.0103 (9)	0.0085 (8)	0.0076 (8)

### Geometric parameters (Å, º)

**Table:**

Ni1—N5	2.022 (2)	C25—N4	1.516 (3)
Ni1—N2	2.024 (2)	C25—H25A	0.9900
Ni1—N6	2.123 (2)	C25—H25B	0.9900
Ni1—N1	2.135 (2)	C26—C31	1.390 (3)
Ni1—O1	2.0277 (16)	C26—C27	1.402 (3)
Ni1—O2	2.0543 (16)	C27—C28	1.391 (3)
C1—N1	1.344 (3)	C27—H27	0.9500
C1—C2	1.381 (4)	C28—C29	1.394 (4)
C1—H1	0.9500	C28—H28	0.9500
C2—C3	1.390 (4)	C29—C30	1.386 (4)
C2—H2	0.9500	C29—H29	0.9500
C3—C4	1.387 (4)	C30—C31	1.389 (4)
C3—H3	0.9500	C30—H30	0.9500
C4—C5	1.384 (3)	C31—H31	0.9500
C4—H4	0.9500	C32—C33	1.490 (3)
C5—N1	1.352 (3)	C32—N4	1.517 (3)
C5—C6	1.511 (3)	C32—H32A	0.9900
C6—N2	1.465 (3)	C32—H32B	0.9900
C6—H6A	0.9900	C33—C38	1.381 (3)
C6—H6B	0.9900	C33—C34	1.424 (3)
C7—N2	1.288 (3)	C34—O2	1.300 (3)
C7—C8	1.451 (3)	C34—C35	1.432 (3)
C7—H7	0.9500	C35—C36	1.417 (3)
C8—C9	1.415 (3)	C35—C40	1.445 (3)
C8—C13	1.429 (3)	C36—C37	1.368 (4)
C9—C10	1.377 (4)	C36—H36	0.9500
C9—H9	0.9500	C37—C38	1.413 (3)
C10—C11	1.405 (3)	C37—C39	1.513 (3)
C10—C14	1.516 (3)	C38—H38	0.9500
C11—C12	1.388 (3)	C39—H39A	0.9800
C11—H11	0.9500	C39—H39B	0.9800
C12—C13	1.429 (3)	C39—H39C	0.9800
C12—C15	1.516 (3)	C40—N5	1.290 (3)
C13—O1	1.305 (3)	C40—H40	0.9500
C14—H14A	0.9800	C41—N5	1.466 (3)
C14—H14B	0.9800	C41—C42	1.513 (3)
C14—H14C	0.9800	C41—H41A	0.9900
C15—N3	1.482 (3)	C41—H41B	0.9900
C15—H15A	0.9900	C42—N6	1.346 (3)
C15—H15B	0.9900	C42—C43	1.386 (3)
C16—N3	1.477 (3)	C43—C44	1.377 (4)
C16—C17	1.513 (3)	C43—H43	0.9500
C16—H16A	0.9900	C44—C45	1.386 (4)
C16—H16B	0.9900	C44—H44	0.9500
C17—C22	1.382 (4)	C45—C46	1.385 (4)
C17—C18	1.395 (4)	C45—H45	0.9500
C18—C19	1.397 (4)	C46—N6	1.345 (3)
C18—H18	0.9500	C46—H46	0.9500
C19—C20	1.364 (5)	C47—O3	1.428 (4)
C19—H19	0.9500	C47—H47A	0.9800
C20—C21	1.390 (5)	C47—H47B	0.9800
C20—H20	0.9500	C47—H47C	0.9800
C21—C22	1.402 (4)	C48—O8	1.399 (4)
C21—H21	0.9500	C48—H48A	0.9800
C22—H22	0.9500	C48—H48B	0.9800
C23—N3	1.458 (3)	C48—H48C	0.9800
C23—C24	1.521 (3)	Cl1—O7	1.434 (2)
C23—H23A	0.9900	Cl1—O5	1.4398 (19)
C23—H23B	0.9900	Cl1—O6	1.4438 (18)
C24—N4	1.496 (3)	Cl1—O4	1.4442 (19)
C24—H24A	0.9900	N4—H4A	0.81 (3)
C24—H24B	0.9900	O3—H3A	0.8400
C25—C26	1.506 (3)	O8—H8	0.8400
			
N5—Ni1—N2	175.37 (8)	C27—C26—C25	120.9 (2)
N5—Ni1—O1	93.85 (7)	C28—C27—C26	120.3 (2)
N2—Ni1—O1	89.25 (7)	C28—C27—H27	119.9
N5—Ni1—O2	89.25 (7)	C26—C27—H27	119.9
N2—Ni1—O2	94.31 (7)	C27—C28—C29	119.9 (2)
O1—Ni1—O2	87.81 (6)	C27—C28—H28	120.0
N5—Ni1—N6	79.90 (8)	C29—C28—H28	120.0
N2—Ni1—N6	96.63 (8)	C30—C29—C28	119.7 (2)
O1—Ni1—N6	90.99 (7)	C30—C29—H29	120.1
O2—Ni1—N6	168.98 (7)	C28—C29—H29	120.1
N5—Ni1—N1	97.29 (8)	C29—C30—C31	120.5 (2)
N2—Ni1—N1	79.67 (8)	C29—C30—H30	119.7
O1—Ni1—N1	168.84 (7)	C31—C30—H30	119.7
O2—Ni1—N1	91.67 (7)	C30—C31—C26	120.2 (2)
N6—Ni1—N1	91.61 (7)	C30—C31—H31	119.9
N1—C1—C2	123.1 (2)	C26—C31—H31	119.9
N1—C1—H1	118.4	C33—C32—N4	108.75 (18)
C2—C1—H1	118.4	C33—C32—H32A	109.9
C1—C2—C3	118.6 (2)	N4—C32—H32A	109.9
C1—C2—H2	120.7	C33—C32—H32B	109.9
C3—C2—H2	120.7	N4—C32—H32B	109.9
C4—C3—C2	118.7 (2)	H32A—C32—H32B	108.3
C4—C3—H3	120.7	C38—C33—C34	122.2 (2)
C2—C3—H3	120.7	C38—C33—C32	121.9 (2)
C5—C4—C3	119.6 (2)	C34—C33—C32	115.9 (2)
C5—C4—H4	120.2	O2—C34—C33	118.3 (2)
C3—C4—H4	120.2	O2—C34—C35	125.5 (2)
N1—C5—C4	121.8 (2)	C33—C34—C35	116.1 (2)
N1—C5—C6	116.5 (2)	C36—C35—C34	119.3 (2)
C4—C5—C6	121.7 (2)	C36—C35—C40	117.0 (2)
N2—C6—C5	110.61 (19)	C34—C35—C40	123.3 (2)
N2—C6—H6A	109.5	C37—C36—C35	123.6 (2)
C5—C6—H6A	109.5	C37—C36—H36	118.2
N2—C6—H6B	109.5	C35—C36—H36	118.2
C5—C6—H6B	109.5	C36—C37—C38	117.0 (2)
H6A—C6—H6B	108.1	C36—C37—C39	122.6 (2)
N2—C7—C8	124.9 (2)	C38—C37—C39	120.5 (2)
N2—C7—H7	117.6	C33—C38—C37	121.5 (2)
C8—C7—H7	117.6	C33—C38—H38	119.3
C9—C8—C13	119.6 (2)	C37—C38—H38	119.3
C9—C8—C7	116.8 (2)	C37—C39—H39A	109.5
C13—C8—C7	123.6 (2)	C37—C39—H39B	109.5
C10—C9—C8	122.9 (2)	H39A—C39—H39B	109.5
C10—C9—H9	118.5	C37—C39—H39C	109.5
C8—C9—H9	118.5	H39A—C39—H39C	109.5
C9—C10—C11	117.3 (2)	H39B—C39—H39C	109.5
C9—C10—C14	122.6 (2)	N5—C40—C35	125.3 (2)
C11—C10—C14	120.1 (2)	N5—C40—H40	117.4
C12—C11—C10	122.0 (2)	C35—C40—H40	117.4
C12—C11—H11	119.0	N5—C41—C42	110.52 (19)
C10—C11—H11	119.0	N5—C41—H41A	109.5
C11—C12—C13	121.0 (2)	C42—C41—H41A	109.5
C11—C12—C15	121.4 (2)	N5—C41—H41B	109.5
C13—C12—C15	117.3 (2)	C42—C41—H41B	109.5
O1—C13—C8	124.0 (2)	H41A—C41—H41B	108.1
O1—C13—C12	119.3 (2)	N6—C42—C43	122.5 (2)
C8—C13—C12	116.6 (2)	N6—C42—C41	117.1 (2)
C10—C14—H14A	109.5	C43—C42—C41	120.3 (2)
C10—C14—H14B	109.5	C44—C43—C42	118.8 (2)
H14A—C14—H14B	109.5	C44—C43—H43	120.6
C10—C14—H14C	109.5	C42—C43—H43	120.6
H14A—C14—H14C	109.5	C43—C44—C45	119.4 (2)
H14B—C14—H14C	109.5	C43—C44—H44	120.3
N3—C15—C12	107.57 (17)	C45—C44—H44	120.3
N3—C15—H15A	110.2	C46—C45—C44	118.4 (2)
C12—C15—H15A	110.2	C46—C45—H45	120.8
N3—C15—H15B	110.2	C44—C45—H45	120.8
C12—C15—H15B	110.2	N6—C46—C45	122.8 (2)
H15A—C15—H15B	108.5	N6—C46—H46	118.6
N3—C16—C17	115.5 (2)	C45—C46—H46	118.6
N3—C16—H16A	108.4	O3—C47—H47A	109.5
C17—C16—H16A	108.4	O3—C47—H47B	109.5
N3—C16—H16B	108.4	H47A—C47—H47B	109.5
C17—C16—H16B	108.4	O3—C47—H47C	109.5
H16A—C16—H16B	107.5	H47A—C47—H47C	109.5
C22—C17—C18	118.2 (2)	H47B—C47—H47C	109.5
C22—C17—C16	120.6 (2)	O8—C48—H48A	109.5
C18—C17—C16	121.1 (2)	O8—C48—H48B	109.5
C17—C18—C19	121.2 (3)	H48A—C48—H48B	109.5
C17—C18—H18	119.4	O8—C48—H48C	109.5
C19—C18—H18	119.4	H48A—C48—H48C	109.5
C20—C19—C18	119.8 (3)	H48B—C48—H48C	109.5
C20—C19—H19	120.1	O7—Cl1—O5	110.50 (13)
C18—C19—H19	120.1	O7—Cl1—O6	109.13 (12)
C19—C20—C21	120.3 (3)	O5—Cl1—O6	109.08 (12)
C19—C20—H20	119.9	O7—Cl1—O4	109.83 (12)
C21—C20—H20	119.9	O5—Cl1—O4	109.34 (12)
C20—C21—C22	119.7 (3)	O6—Cl1—O4	108.93 (11)
C20—C21—H21	120.2	C1—N1—C5	118.2 (2)
C22—C21—H21	120.2	C1—N1—Ni1	127.39 (16)
C17—C22—C21	120.9 (3)	C5—N1—Ni1	114.12 (16)
C17—C22—H22	119.6	C7—N2—C6	117.5 (2)
C21—C22—H22	119.6	C7—N2—Ni1	126.80 (16)
N3—C23—C24	110.21 (19)	C6—N2—Ni1	115.58 (15)
N3—C23—H23A	109.6	C23—N3—C16	114.08 (19)
C24—C23—H23A	109.6	C23—N3—C15	112.07 (18)
N3—C23—H23B	109.6	C16—N3—C15	115.56 (18)
C24—C23—H23B	109.6	C24—N4—C25	114.26 (18)
H23A—C23—H23B	108.1	C24—N4—C32	111.68 (17)
N4—C24—C23	109.03 (18)	C25—N4—C32	112.75 (17)
N4—C24—H24A	109.9	C24—N4—H4A	106 (2)
C23—C24—H24A	109.9	C25—N4—H4A	109 (2)
N4—C24—H24B	109.9	C32—N4—H4A	102 (2)
C23—C24—H24B	109.9	C40—N5—C41	116.9 (2)
H24A—C24—H24B	108.3	C40—N5—Ni1	127.53 (17)
C26—C25—N4	112.55 (18)	C41—N5—Ni1	115.57 (15)
C26—C25—H25A	109.1	C46—N6—C42	118.0 (2)
N4—C25—H25A	109.1	C46—N6—Ni1	127.74 (17)
C26—C25—H25B	109.1	C42—N6—Ni1	114.22 (15)
N4—C25—H25B	109.1	C13—O1—Ni1	123.48 (14)
H25A—C25—H25B	107.8	C34—O2—Ni1	126.59 (15)
C31—C26—C27	119.3 (2)	C47—O3—H3A	109.5
C31—C26—C25	119.8 (2)	C48—O8—H8	109.5
			
N1—C1—C2—C3	0.2 (4)	N5—Ni1—N1—C1	13.0 (2)
C1—C2—C3—C4	−0.1 (4)	N2—Ni1—N1—C1	−170.6 (2)
C2—C3—C4—C5	0.0 (4)	O1—Ni1—N1—C1	−163.6 (3)
C3—C4—C5—N1	0.0 (4)	O2—Ni1—N1—C1	−76.50 (19)
C3—C4—C5—C6	178.2 (2)	N6—Ni1—N1—C1	93.0 (2)
N1—C5—C6—N2	−18.1 (3)	N5—Ni1—N1—C5	−173.16 (16)
C4—C5—C6—N2	163.7 (2)	N2—Ni1—N1—C5	3.31 (16)
N2—C7—C8—C9	−172.8 (2)	O1—Ni1—N1—C5	10.2 (4)
N2—C7—C8—C13	5.0 (4)	O2—Ni1—N1—C5	97.39 (16)
C13—C8—C9—C10	−2.9 (3)	N6—Ni1—N1—C5	−93.14 (16)
C7—C8—C9—C10	175.1 (2)	C8—C7—N2—C6	179.0 (2)
C8—C9—C10—C11	−2.6 (3)	C8—C7—N2—Ni1	3.4 (3)
C8—C9—C10—C14	179.3 (2)	C5—C6—N2—C7	−155.3 (2)
C9—C10—C11—C12	2.6 (3)	C5—C6—N2—Ni1	20.8 (2)
C14—C10—C11—C12	−179.3 (2)	N5—Ni1—N2—C7	−148.8 (9)
C10—C11—C12—C13	2.9 (3)	O1—Ni1—N2—C7	−16.6 (2)
C10—C11—C12—C15	−171.0 (2)	O2—Ni1—N2—C7	71.1 (2)
C9—C8—C13—O1	−171.1 (2)	N6—Ni1—N2—C7	−107.5 (2)
C7—C8—C13—O1	11.1 (3)	N1—Ni1—N2—C7	162.1 (2)
C9—C8—C13—C12	8.0 (3)	N5—Ni1—N2—C6	35.5 (10)
C7—C8—C13—C12	−169.7 (2)	O1—Ni1—N2—C6	167.71 (16)
C11—C12—C13—O1	171.0 (2)	O2—Ni1—N2—C6	−104.54 (16)
C15—C12—C13—O1	−14.8 (3)	N6—Ni1—N2—C6	76.80 (16)
C11—C12—C13—C8	−8.2 (3)	N1—Ni1—N2—C6	−13.63 (16)
C15—C12—C13—C8	166.02 (19)	C24—C23—N3—C16	−78.8 (2)
C11—C12—C15—N3	101.9 (2)	C24—C23—N3—C15	147.50 (19)
C13—C12—C15—N3	−72.3 (2)	C17—C16—N3—C23	−53.1 (3)
N3—C16—C17—C22	101.3 (3)	C17—C16—N3—C15	79.0 (3)
N3—C16—C17—C18	−75.1 (3)	C12—C15—N3—C23	−76.3 (2)
C22—C17—C18—C19	−2.0 (4)	C12—C15—N3—C16	150.7 (2)
C16—C17—C18—C19	174.5 (3)	C23—C24—N4—C25	−78.5 (2)
C17—C18—C19—C20	1.3 (5)	C23—C24—N4—C32	152.04 (19)
C18—C19—C20—C21	−0.3 (5)	C26—C25—N4—C24	−58.1 (2)
C19—C20—C21—C22	0.0 (5)	C26—C25—N4—C32	70.8 (2)
C18—C17—C22—C21	1.8 (4)	C33—C32—N4—C24	−71.9 (2)
C16—C17—C22—C21	−174.8 (2)	C33—C32—N4—C25	157.81 (18)
C20—C21—C22—C17	−0.8 (4)	C35—C40—N5—C41	170.5 (2)
N3—C23—C24—N4	−53.6 (2)	C35—C40—N5—Ni1	−9.5 (3)
N4—C25—C26—C31	98.6 (2)	C42—C41—N5—C40	161.6 (2)
N4—C25—C26—C27	−82.7 (3)	C42—C41—N5—Ni1	−18.4 (3)
C31—C26—C27—C28	−1.4 (3)	N2—Ni1—N5—C40	−124.6 (9)
C25—C26—C27—C28	179.9 (2)	O1—Ni1—N5—C40	103.4 (2)
C26—C27—C28—C29	0.9 (4)	O2—Ni1—N5—C40	15.7 (2)
C27—C28—C29—C30	0.2 (4)	N6—Ni1—N5—C40	−166.3 (2)
C28—C29—C30—C31	−0.9 (4)	N1—Ni1—N5—C40	−75.9 (2)
C29—C30—C31—C26	0.3 (4)	N2—Ni1—N5—C41	55.4 (10)
C27—C26—C31—C30	0.8 (4)	O1—Ni1—N5—C41	−76.60 (17)
C25—C26—C31—C30	179.6 (2)	O2—Ni1—N5—C41	−164.35 (17)
N4—C32—C33—C38	115.9 (2)	N6—Ni1—N5—C41	13.72 (17)
N4—C32—C33—C34	−62.0 (2)	N1—Ni1—N5—C41	104.06 (17)
C38—C33—C34—O2	177.4 (2)	C45—C46—N6—C42	0.4 (4)
C32—C33—C34—O2	−4.7 (3)	C45—C46—N6—Ni1	177.01 (19)
C38—C33—C34—C35	−5.8 (3)	C43—C42—N6—C46	−1.6 (3)
C32—C33—C34—C35	172.07 (19)	C41—C42—N6—C46	174.6 (2)
O2—C34—C35—C36	−177.2 (2)	C43—C42—N6—Ni1	−178.64 (19)
C33—C34—C35—C36	6.2 (3)	C41—C42—N6—Ni1	−2.5 (3)
O2—C34—C35—C40	10.9 (4)	N5—Ni1—N6—C46	177.2 (2)
C33—C34—C35—C40	−165.6 (2)	N2—Ni1—N6—C46	0.3 (2)
C34—C35—C36—C37	−1.8 (3)	O1—Ni1—N6—C46	−89.1 (2)
C40—C35—C36—C37	170.6 (2)	O2—Ni1—N6—C46	−172.7 (3)
C35—C36—C37—C38	−3.4 (4)	N1—Ni1—N6—C46	80.0 (2)
C35—C36—C37—C39	177.4 (2)	N5—Ni1—N6—C42	−6.09 (16)
C34—C33—C38—C37	0.7 (4)	N2—Ni1—N6—C42	177.00 (16)
C32—C33—C38—C37	−177.0 (2)	O1—Ni1—N6—C42	87.64 (16)
C36—C37—C38—C33	4.0 (4)	O2—Ni1—N6—C42	4.0 (5)
C39—C37—C38—C33	−176.8 (2)	N1—Ni1—N6—C42	−103.21 (17)
C36—C35—C40—N5	−178.9 (2)	C8—C13—O1—Ni1	−32.7 (3)
C34—C35—C40—N5	−6.8 (4)	C12—C13—O1—Ni1	148.21 (17)
N5—C41—C42—N6	13.3 (3)	N5—Ni1—O1—C13	−152.90 (17)
N5—C41—C42—C43	−170.4 (2)	N2—Ni1—O1—C13	30.54 (17)
N6—C42—C43—C44	1.8 (4)	O2—Ni1—O1—C13	−63.80 (17)
C41—C42—C43—C44	−174.3 (2)	N6—Ni1—O1—C13	127.16 (17)
C42—C43—C44—C45	−0.8 (4)	N1—Ni1—O1—C13	23.7 (4)
C43—C44—C45—C46	−0.3 (4)	C33—C34—O2—Ni1	178.09 (14)
C44—C45—C46—N6	0.5 (4)	C35—C34—O2—Ni1	1.6 (3)
C2—C1—N1—C5	−0.2 (3)	N5—Ni1—O2—C34	−11.76 (18)
C2—C1—N1—Ni1	173.47 (18)	N2—Ni1—O2—C34	165.27 (17)
C4—C5—N1—C1	0.1 (3)	O1—Ni1—O2—C34	−105.65 (17)
C6—C5—N1—C1	−178.2 (2)	N6—Ni1—O2—C34	−21.7 (4)
C4—C5—N1—Ni1	−174.42 (18)	N1—Ni1—O2—C34	85.50 (18)
C6—C5—N1—Ni1	7.4 (3)		

### Hydrogen-bond geometry (Å, º)

**Table:**

*D*—H···*A*	*D*—H	H···*A*	*D*···*A*	*D*—H···*A*
N4—H4*A*···O2	0.81 (3)	2.21 (3)	2.829 (3)	134 (3)
N4—H4*A*···N3	0.81 (3)	2.35 (3)	2.809 (3)	117 (2)
C45—H45···O8^i^	0.95	2.52	3.417 (3)	156
C41—H41*A*···O6	0.99	2.34	3.276 (3)	157
C31—H31···O8^ii^	0.95	2.43	3.365 (3)	167
C30—H30···O5^ii^	0.95	2.58	3.524 (3)	171
C28—H28···O6^iii^	0.95	2.56	3.503 (3)	173
C7—H7···O6^i^	0.95	2.60	3.289 (3)	130
C4—H4···O4^iv^	0.95	2.49	3.405 (3)	163
O8—H8···O3^v^	0.84	1.91	2.748 (3)	176
O3—H3*A*···O1^vi^	0.84	1.91	2.723 (3)	164

Symmetry codes: (i) *x*+1, *y*, *z*; (ii) *x*+1, *y*−1, *z*; (iii) −*x*+2, −*y*, −*z*+1; (iv) −*x*+2, −*y*+1, −*z*+1; (v) −*x*, −*y*+1, −*z*; (vi) *x*−1, *y*, *z*.
